# Metagenomic Sequencing of Two Cultures Grown on Chemically Deconstructed Plastic Products

**DOI:** 10.1128/mra.01304-22

**Published:** 2023-06-20

**Authors:** Lindsay I. Putman, Laura G. Schaerer, Ruochen Wu, Daniel G. Kulas, Ali Zolghadr, Rebecca G. Ong, David R. Shonnard, Stephen M. Techtmann, Lucille G. Arbanas, Gabriel G. Bannerman, Brice Cart, Allen Cureton, Benjamin P. Doerr, Zora Jovicevic, Mary Langosch, Aaron B. MacLeod, Caden McCloskey, Avery M. McNally, Mykenzie K. Monkevich, Adrian Noecker, Dylan Norris, Vincent G. Pellizzon, Kylie B. Strom, Emily E. Taylor

**Affiliations:** a Department of Biological Sciences, Michigan Technological University, Houghton, Michigan, USA; b Department of Chemical Engineering, Michigan Technological University, Houghton, Michigan, USA; c Department of Chemistry, Michigan Technological University, Houghton, Michigan, USA; University of Southern California

## Abstract

We report the metagenome sequences of two microbial cultures that were grown with chemically deconstructed plastic products as their sole carbon source. These metagenomes will provide insights into the metabolic capabilities of cultures grown on deconstructed plastics and can serve as a starting point for the identification of novel plastic degradation mechanisms.

## ANNOUNCEMENT

Over one-half of the world’s plastic is discarded into landfills, where degradation takes tens to thousands of years (1, 2). We explored the capacity of microbial consortia to degrade chemically deconstructed polycarbonate (PC) and polyethylene terephthalate (PET) and thermally deconstructed high-density polyethylene (HDPE) and low-density polyethylene (LDPE) (2, 3). Metagenomes from two cultures grown using real and anticipated depolymerization products from these materials are reported here.

Five grams of PC and 175 mL of ammonium hydroxide (28 to 30% by weight solution in water) were added to a custom batch reactor and heated to 120°C for 30 min. The product was filtered and neutralized using phosphoric acid. HDPE dissolved in paraffin wax solvent was fed into a custom reactor and heated to 575°C (vapor residence time, ~4 s) to produce pyrolysis oil, as described previously (3, 4).

The LIP and SMT cultures are separate enrichments that were inoculated with vermicompost from a farm in Calumet, Michigan, USA (coordinates: 47.211, −88.553), and grown in 90 mL of Bushnell-Haas broth. The LIP culture was amended with 0.25 g of disodium terephthalate, 0.25 g of terephthalamide, 2.5 mL of chemically deconstructed PC, and 0.25 mL of a 1:1:1:1 mixture of C_6_:C_10_:C_16_:C_20_ alkenes. The SMT culture was amended with 1 g of disodium terephthalate and 0.74 mL of HDPE pyrolysis oil. Cultures were grown aerobically at 30°C in a shaking incubator. Ten milliliters of culture was transferred to 90 mL of fresh medium every 14 days (LIP) or 7 days (SMT). After growth for 10 weeks (LIP) or 7 weeks (SMT), 40 mL of culture was harvested for DNA extraction using the FastDNA Spin kit for soil (MP Biomedicals).

The University of Utah High-Throughput Genomics Core Facility (UU-HTGCF) used the Nextera Flex DNA library preparation kit (Illumina) for DNA fragmentation and library preparation and normalization. Libraries were evaluated using a Bioanalyzer DNA 1000 chip kit (Agilent Technologies). Paired-end sequencing (2 × 150 bp) was performed on an Illumina NovaSeq 6000 system with a NovaSeq S4 reagent kit v1.5 (Illumina). Libraries were multiplexed and pooled into one lane. After demultiplexing and quality assessment were performed by the UU-HTGCF, 88,071,978 raw reads (LIP) and 112,316,479 raw reads (SMT) were obtained. The average read length was 151 bp for both metagenomes.

Default parameters were used for all analyses unless otherwise noted. Trimmomatic v0.39 (5) was used to quality filter sequences, which resulted in 87,009,321 paired reads and 110,892,861 paired reads from the LIP and SMT metagenomes, respectively. Trimmed reads were assembled using metaSPAdes v3.14.1 (6) with kmer sizes of 21, 51, 71, 91, 111, and 127. Assembly quality was assessed using metaQUAST v5.0.2 (7). Contigs were annotated using Prokka v1.14.6 (8). Table 1 shows statistics for the metagenome assemblies and annotations. GhostKOALA v2.2 (9) was used to annotate protein sequences obtained from Prokka and further assess metabolic pathways using KEGG Mapper Reconstruct v5.0 (10). Contigs were not binned.

**TABLE 1:** Quality information and features of the assembled metagenomic contigs

Feature	Finding for:	
	LIP metagenome	SMT metagenome
Total assembly length (bp)	48,618,614	69,768,209
Length of longest contig (bp)	1,119,130	2,386,692
No. of contigs	11,805	20,514
*N*_50_ (bp)	119,442	141,331
*L*_50_	91	88
No. of coding sequences	45,977	63,564
No. of rRNAs	36	53
No. of CRISPRs	3	5
No. of tRNAs	520	688

Annotation of the metagenomes predicted genes and pathways relevant to the degradation of aliphatic and aromatic hydrocarbons that are depolymerization products of HDPE, LDPE, PC, and PET, including phthalate degradation, protocatechuate degradation, bisphenol A degradation, alkane degradation, and beta oxidation (11–16).

### Data availability.

Data are available under NCBI BioProject accession number PRJNA849162, Sequence Read Archive (SRA) accession numbers SRR22262629 and SRR22689345, assembly accession numbers GCA_029289365.1 and GCA_029289405.1, and BioSample accession numbers SAMN29041702 and SAMN32170191. Prokka and GhostKOALA annotation outputs are available at figshare (https://figshare.com/projects/FS22_BL2700_MRA_Metagenomes/161494).
